# JPHYD Inhibits miR-21-5p/Smad7-Mediated Epithelial-Mesenchymal Transition of Hepatocellular Carcinoma Cells

**DOI:** 10.1155/2022/7823433

**Published:** 2022-04-26

**Authors:** Li-Hua Liu, Chong-Kai Fang, Fu-Cheng Ge, Ji-Nan Wang, Xiu-Bing Zhang, Rui Luo, Ying Zhang, Kun-Liang Feng, Zhen-Wen Qiu, Chong Zhong

**Affiliations:** ^1^Department of Hepatobiliary Surgery, The First Affiliated Hospital of Guangzhou University of Chinese Medicine, Guangzhou 510405, China; ^2^Lingnan Medical Research Center, Guangzhou University of Chinese Medicine, Guangzhou 510405, China; ^3^First Clinical Medical College, Guangzhou University of Chinese Medicine, Guangzhou 510405, China; ^4^Department of Pharmacy, The First Affiliated Hospital of Guangzhou University of Chinese Medicine, Guangzhou 510405, China

## Abstract

**Background:**

Studies have shown that *Jianpi Huayu Decoction* (JPHYD) can inhibit the growth of hepatocellular carcinoma cells, but the mechanism of its effect was not clear at present.

**Methods:**

We assessed the effect of JPHYD using liver cancer cells as in vitro cell model and xenograft tumor as in vivo model. CCK8, EdU, wound-healing, and transwell assays were performed to assess the cell growth, migration, and invasion of hepatocellular carcinoma (HCC) cell lines HepG2 and MHCC97H. Western blot assay was performed to observe the protein level of E-cadherin, Smad7, N-cadherin, Snail, Smad3, Vimentin, and Zeb1. qRT-PCR assay was used to observe the expression of miR-21-5p in clinical liver cancer tissue samples and in HepG2 and MHCC97H cells. Animal tumorigenesis experiments and in vivo imaging experiments were performed to assess the results of in vitro experiments.

**Results:**

We found that JPHYD could inhibit the proliferation, invasion, and migration of hepatocellular carcinoma cells and JPHYD decreased the level of N-cadherin, Snail, Vimentin, Smad3, and Zeb1 and increased E-cadherin and Smad7 proteins. The expression of miR-21-5p was increased while that protein of Smad7 was decreased in HCC tissues. The vivo experiments also showed that miR-21-5p could promote the migration of HCC cells. JPHYD decreased miR-21-5p expression. The same results have been found in animal studies.

**Conclusion:**

Our results indicated that JPHYD inhibited epithelial-mesenchymal transition by increasing Smad7 expression and inhibiting miR-21-5p. Therefore, blocking the occurrence and development of EMT may be a new mechanism of JPHYD's anti-liver cancer effect.

## 1. Introduction

Liver cancer ranks the sixth and the third in incidence and mortality worldwide [1]. The occurrence of liver cancer is insidious, and the tumor grows extremely fast. When symptoms appear, it is often in the advanced stage of the tumor [2]. Although targeted therapy and chemotherapy can significantly shrink tumors, some patients cannot tolerate the toxicity of the drugs, and others are prone to drug resistance, which severely limits the survival time and quality of life of liver cancer patients [3, 4].

Tumor metastasis is the main reason that affects the treatment and survival rate of patients [5]. Metastatic tumors are usually highly invasive; therefore, new treatments to curb the malignant pathological characteristics of metastatic tumors are widely explored. Epithelial-mesenchymal transition (EMT), a process of epithelial cell phenotypic transformation to mesenchymal cell, is the initial step of tumor metastasis which promotes the metastatic ability of tumor cells [6, 7]. Thus, it is crucial to prevent tumor cells from developing EMT to block tumor metastasis.

Oncologists of Traditional Chinese Medicine (TCM) have been working to develop novel treatments that can increase the antitumor effect with few toxic effects. Many studies show that TCM can effectively improve immunity, prevent, or cure the occurrence and development of malignant tumors. In addition, the combined treatment of TCM and western medicine has a synergistic effect which can reduce the toxic and side effects of western medicine treatment, improving the quality of life of patients.

*Jianpi Huayu Decoction* (JPHYD) is a Chinese prescription for treating malignant liver cancer, which has a widespread application in clinics for nearly 10 years. The ingredients of JPHYD are *Ginseng Radix et Rhizoma* (Renshen, dry roots and rhizomes of Panax ginseng C.A. Mey.), *Atractylodis Macrocephalae Rhizoma* (Baizhu, dry rhizomes of *Atractylodes macrocephala* Koidz.), *Dioscoreae Rhizoma* (Shanyao, dry rhizomes of *Dioscorea opposita* Thunb.), *Poria* (Fuling, dry sclerotia of Poria cocos (Schw.) Wolf), *Moutan Cortex* (Mudanpi, dry root bark of *Paeonia suffruticosa* Andr.), *Salviae Miltiorrhizae Radix et Rhizoma* (Danshen, dry roots and rhizomes of *Salvia miltiorrhiza* Bge.), *Curcumae Radix* (Yujin, dry root tuber of *Curcuma longa* L.), *Curcumae Rhizoma* (Ezhu, dry rhizomes of *C. wenyujin* Y. H. Chen et C. Ling), *Bupleuri Radix* (Chaihu, dry root of Bupleurum chinense DC), and *Glycyrrhizae Radix et Rhizoma* (Gancao, dry roots and rhizomes of Glycyrrhiza uralensis Fisch.). All of the components are recorded in the *Chinese Pharmacopoeia.* Our research team has found that JPHYD combined with TACE (transhepatic arterial chemotherapy and embolization) can effectively prolong the survival time of patients and improve their quality of life [8]. Meanwhile, we also found that JPHYD can inhibit the proliferation and invasion of HCC cells through Smads and EMT signaling pathways [9]. However, the mechanism of JPHYD in regulating EMT signaling pathway is not clear. In this study, we found that JPHYD can inhibit EMT of hepatocellular carcinoma (HCC) cells through miR-21-5p/smad7 signaling pathway both in vivo and in vitro.

## 2. Materials and Methods

### 2.1. Cell Culture and Reagents

HCC cell lines MHCC97H and HepG2 were cultured in Dulbecco's Modified Eagle Medium (DMEM) (Gibco, Grand Island, NY, USA) with 100 U/mL penicillin and streptomycin (Gibco, Grand Island, NY, USA) and 10% heat-inactivated fetal bovine serum (FBS), and then the cells were cultured in a humidified environment with a temperature of 37 °C and 5% carbon dioxide (CO2). JPHYD medicinal materials were purchased from the First Affiliated Hospital of Guangzhou University of Chinese Medicine and dissolved in DMEM.

### 2.2. Cell Viability

The growth of MHCC97H and HepG2 cells was tested by Cell Counting Kit-8 (GLPBIO, USA) experiment (CCK-8). Cells were cultured in a 96-well plate, different concentrations of JPHYD (0, 1, 2, 4, 6, 8, and10 mg/mL) were added to the cells for 24-72 h, then 10 *μ*l of CCK-8 reagent was added to the cells for 2 h, and finally the microplate reader (BioRad, Hercules, CA, USA) was used to measure the absorbance at 450 nm.

### 2.3. EdU Incorporation Assay

The cell proliferation assay was detected by the Cell-Light EdU In Vitro Kit (RiboBio, China). After adding 50 *μ*mol/L 5-ethynyl-2′-deoxyuridine (EdU) to the cells for 2 hours and then staining them with ApolloDye Solution and Hoechst 33342 for 30 min, we placed the cells under a microscope to take pictures. The assessment of cell proliferation capacity was based on the percentage of Edu positive cells.

### 2.4. Wound Healing Assay

Cells were put into the 6-well plate (4000 cells/well). After the cells grew to 80%, we scratched the cells with a sterile pipette tip. After washing the cells with PBS, different concentrations of JPHYD were added to the cells, and then the cells were placed under a microscope to take a 0 h pictures. 24 hours later, the cells were taken pictures again.

### 2.5. Invasion Assay

Cells were cultured in a transwell plate, and the corresponding concentration of JPHYD was added to the cells to culture for 24 hours. Then, according to the instructions, the cells were fixed with 4% paraformaldehyde. After that, the cells were stained with 0.5% crystal violet solution (Beyotime, China). The invasion ability of a cell was measured by the number of cell invasion.

### 2.6. Western Blot Analysis

After lysising MHCC97H and HepG2 cells with RIPA lysis buffer (Beyotime, Shanghai, China) (Beyotime, Shanghai, China), then we separated an equal amount of protein on a 10% sodium dodecyl sulfate (SDS) polyacrylamide gel and transferred the protein to a polyvinylidene fluoride (PVDF) membrane, which was associated with anti-E-cadherin, Smad7, N-cadherin, Vimentin, Smad3, Snail, and Zeb1 (1 : 1000 dilution, Proteintech, Wuhan, China) which were incubated overnight at 4 °C. Then, we incubated the membrane with anti-rabbit IgG and HRP-linked secondary antibody (1 : 3000, Cell Signaling Technology, MA) for 1.5 hours at room temperature, USA). Finally, we used Immobilon® Western chemiluminescent HRP substrate (Millipore, Billerica, MA, USA) for visualization.

### 2.7. Quantitative Real-Time PCR

TRIzol Reagent (GLPBIO, Montclair, CA, USA) was used to extract RNA. The extracted total RNA concentration was determined, and then the RNA was reverse-transcribed into cDNA using a reverse transcription kit (Accurate Biology, Hunan, China). The miRNA qRT-PCR Starter Kit (RiboBio, Guangzhou, China) was used to analysis miR-21-5p level, and U6 was used for the internal control for normalization purposes, and U6 was used as a control. Their sequences were as follows: miR-21-5p: AUCACAUUGCCAGGGAUUUCC; U6: forward: CGCTTCGGCAGCACATATAC; and U6: reverse: TTCACGAATTTGCGTGTCATC. The results were evaluated by using the 2-*ΔΔ*Ct method.

### 2.8. Immunochemistry

Sections were incubated in Hydrogen Peroxide Block for 10-15 minutes. Then, Ultra V Block dropwise was added to block nonspecific background staining at room temperature for 5 minutes. The anti-ki67 (1 : 100, Cell Signaling Technology, MA) was added to the sections, and the sections were incubated overnight at 4 °C. A secondary antibody (1 : 200) was added after washing with PBS and incubated for 1 h at room temperature.

### 2.9. Cell Transfection Assay

PmirGLO-SMAD7 (HedgehogBio, Shanghai, China) was constructed. The miR-21-5p mimic, miR-21-5p inhibitor ,and negative control were designed and synthesized by RiboBio Co, Ltd. (Guangzhou, China). Lipofectamine 3000 reagent was used to transfect them into MHCC97H and HepG2 cells in a 24-well plate.

### 2.10. Dual-Luciferase Reporter Assay

The dual luciferase reporter gene detection kit (Beyotime, China) was used to evaluate the firefly and Renilla luciferase activity 24 hours after transfection. Then, we use Lipofectamine 3000 reagent to transfect MHCC97H and HepG2 cells with Smad7 overexpression plasmid and control vector in a 24-well plate.

### 2.11. Animal Study

The BALB/c nude mice aged 5 weeks were purchased from the First Affiliated Hospital of Guangzhou University of Chinese Medicine. The fluorescent MHCC97H cells were injected into the armpit of nude mice. One week later, the nude mice were divided into three groups; they were the control group, JPHYD (1 g/mL), and JPHYD (2 g/mL). Then, the tumor volume and body weight of nude mice were measured every day. We took pictures under the animal imaging machine to observe the tumor size. After the nude mice died, the tumors were removed, and related experiments were performed.

### 2.12. Clinical Tissues and Ethics Statement

The surgical specimens used in clinical research were approved by the ethics committee of Guangzhou First Clinical Medical College (ethics approval number ZYYECK [2019]008). The patient did not receive radiotherapy or chemotherapy before surgery. The tissues obtained by surgery were used for qRT-PCR to verify and explore the correlation between miR-21-5p and smad7 and the overall survival rate of patients after hepatectomy.

### 2.13. Statistical Analysis

The GraphPad Prism 8.0 software (LaJolla, CA, USA) was used to analyze all data. The data of each experiment were expressed as the mean ± SD. To assess differences between groups, a one-way analysis of variance was used, while for multiple comparisons between specific groups, the Turkish multiple comparison test was used. If *P* < 0.05, the difference was considered significant.

## 3. Results

### 3.1. JPHYD Inhibited the Proliferation of MHCC97H and HepG2 Cells

**Figure 1:**
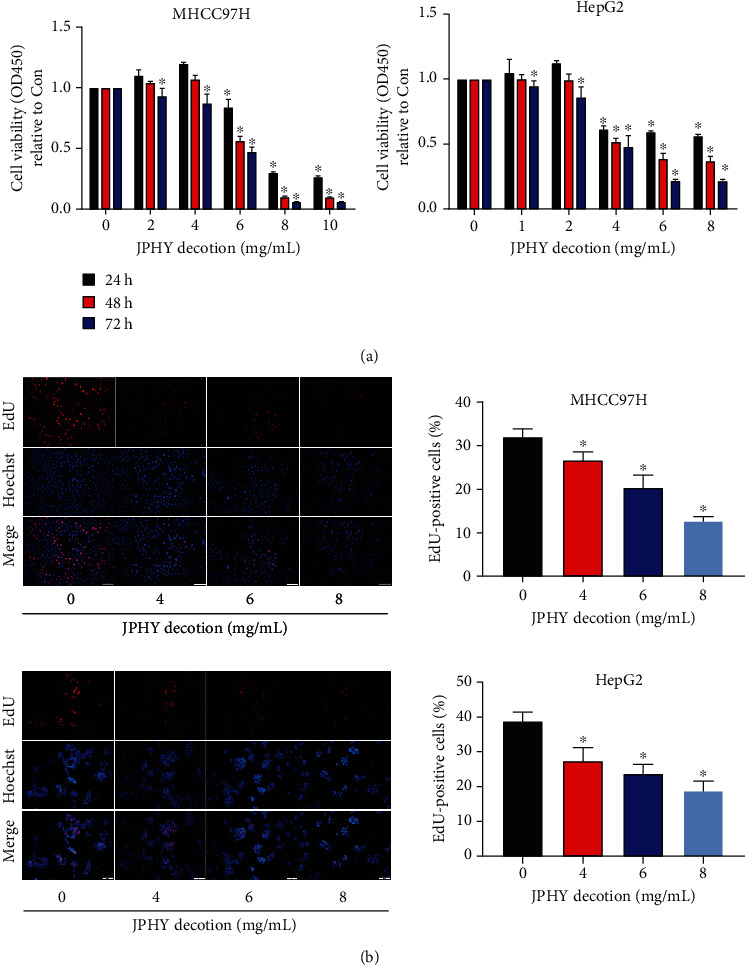
JPHYD inhibited the proliferation of MHCC97H and Hep G2 cells. (a) We used gradual increasing concentration of JPHYD to interfere with liver cancer cells for 24-72 h and observed the cell proliferation by CCK8 experiment. (b) After the CCK8 experiment, the optimal intervention concentration of JPHYD was obtained. The three JPHYD concentrations including the optimal intervention concentration were selected to intervene cells, and the operation was performed according to the EdU kit, and then the positive rate of EdU was calculated. The value given was the mean ± SD from 3 independent experiments performed in triplicate. The asterisk (∗) indicates significantly different from the control group (*P* < 0.05).

CCK8 experiment and EdU experiment were used to detect the proliferation of HCC cells. The CCK8 experiment result showed that JPHYD inhibited the viability of HCC cells (Figure 1(a)). Similarly, in the EdU experiment, we found that JPHYD inhibited cell proliferation (Figure 1(b)). Therefore, it could be proved that these two experiments showed that JPHYD inhibited the proliferation of HCC cells.

### 3.2. JPHYD Reduced Cell Migration and Invasion of MHCC97H and HepG2 Cells

**Figure 2:**
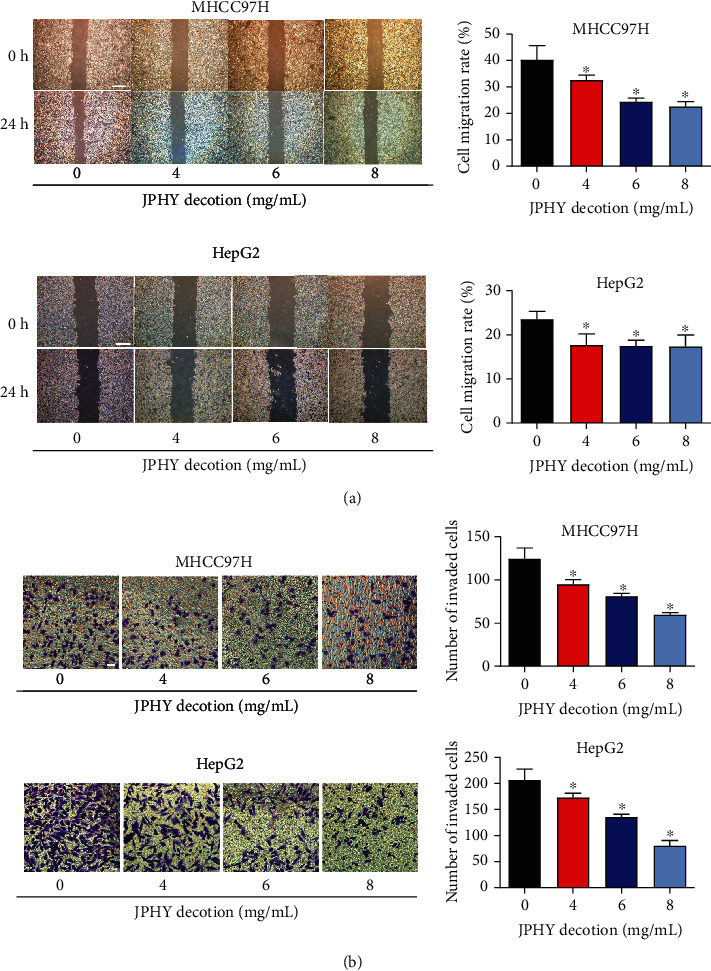
JPHYD inhibited cell migration and invasion of MHCC97H and Hep G2.(a) Cell migration experiment. Scratching the liver cancer cells with a growth density of 80%. After washing the cells with PBS, we added JPHYD to coculture and take pictures. After 24 hours, we took pictures again. (b) Cell invasion experiment. We added cells to the transwell chamber where matrigel has been spread and added the corresponding JPHYD to culture. After 24 h, the cells were fixed, stained, and photographed under a microscope. Finally, we counted the number of cell invasion. Scale bar = 100 *μ*m. The asterisk (∗) indicates a significant difference from the control group (*P* < 0.05).

Next, the role of JPHYD in migration and invasion was determined by wound healing and transwell assays using HepG2 and MHCC97H cells. HepG2 and MHCC97H cells were treated with JPHYD (4, 6, and 8 mg/mL) for 24 hours, and then wound healing and invasion experiments were performed. The results showed that JPHYD significantly inhibited cell migration and invasion ability in HepG2 and MHCC97H cells; however, there is no obvious concentration dependence in cell migration assay (Figures 2(a) and 2(b)). These results indicated that JPHYD inhibited the migration and invasion of HepG2 and MHCC97H cells.

### 3.3. JPHYD Downregulated Level of Smad3, Vimentin, N-Cadherin, Snail, and Zeb1 and Increased the Level of Smad7 and E-Cadherin Proteins

**Figure 3:**
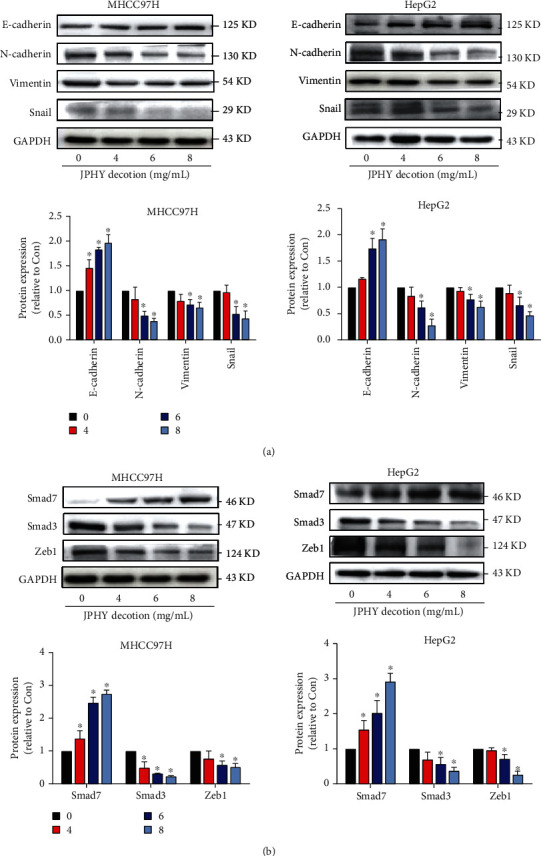
JPHYD inhibited the expression of N-cadherin, Vimentin, Snail, Smad3, and Zeb1 and increased the expression of E-cadherin and Smad7 proteins in MHCC97H and HepG2 cells. (a) Different concentrations of JPHYD were interfered with liver cancer cells for 24 hours, and the expression of E-cadherin, N-cadherin, Vimentin, and Snail proteins and (b) Smad7, Smad3, and Zeb1 proteins measured via Western blot analysis. The asterisk (∗) indicates a significant difference from the control group (*P* < 0.05).

In order to further verify the effect of JPHYD on liver cancer cells, we performed the Western Blot analysis. The results showed that JPHYD significantly inhibited the level of Smad3, Vimentin, N-cadherin, Snail, and Zeb1 and increased the level of Smad7 and E-cadherin proteins (Figures 3(a) and 3(b)). These results revealed that JPHYD inhibited cell growth inhibition and EMT processes by inhibiting N-cadherin, Vimentin, Snail, Smad3, and Zeb1 and promoting E-cadherin and Smad7.

### 3.4. miR-21-5p Promoted the Occurrence of Liver Cancer and the Migration of Liver Cancer Cells

**Figure 4:**
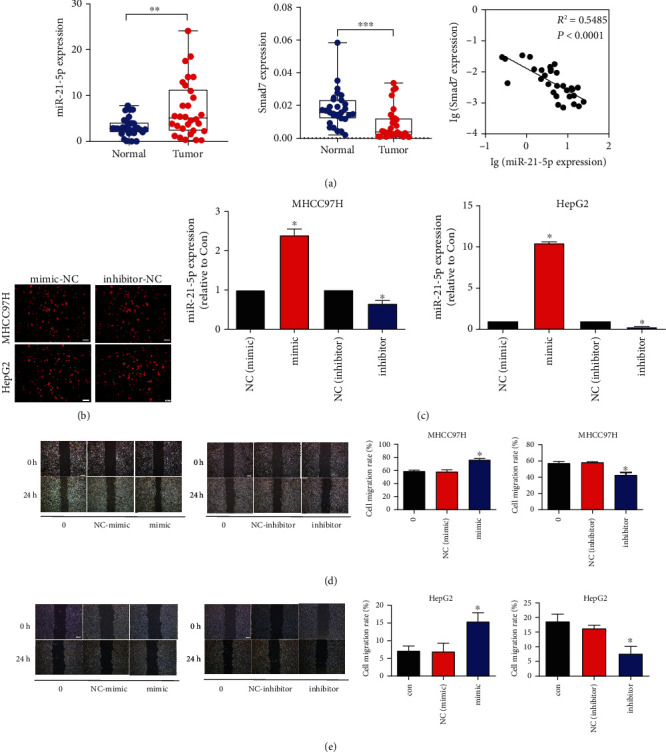
miR-21-5p was highly expressed in liver cancer tissues and liver cancer cells. (a) The expression of miR-21-5p and Smad7 in liver cancer tissues. (b) The transfection of miR-21-5p-NC in MHCC97H and HepG2 cells. (c) The expression of miR-21-5p in MHCC97H and HepG2 cells after miR-21-5p mimic and inhibitor transfection. (d, e) After the transfection of miR-21-5p-NC in MHCC97H and HepG2 cells for 48 hours, wound-healing assays were performed to observe the migration of MHCC97H and HepG2 cells. The asterisk (∗) indicates a significant difference from the control group (*P* < 0.05).

In clinical trials, we found that in the cancer tissues of HCC patients, the expression of Smad7 decreased, while the expression of miR-21-5p increased, indicating that miR-21-5p promoted the development of liver cancer (Figure 4(a)). Therefore, we transfected the mimics of miR-21-5p and miR-21-5p-inhibitors into liver cancer MHCC97H and HepG2 cells to observe whether the proliferation of liver cancer cells was affected. First, we transfected the miR-21-5p-mimic-NC and miR-21-5p-inhibitor-NC with red fluorescence into the cells and observed the transfection of the cells (Figure 4(b)). Then, we performed PCR experiments on the transfected cells to observe the level of miR-21-5p (Figure 4(c)). Finally, we observed the effect of miR-21-5p on the migration of liver cancer cells through a wound-healing test. Experimental results showed that miR-21-5p promoted the migration of liver cancer cells (Figures 4(d) and 4(e)).

### 3.5. JPHYD Increased the Expression of Smad7 and Decreased the Level of miR-21-5p in MHCC97H and HepG2 Cells

**Figure 5:**
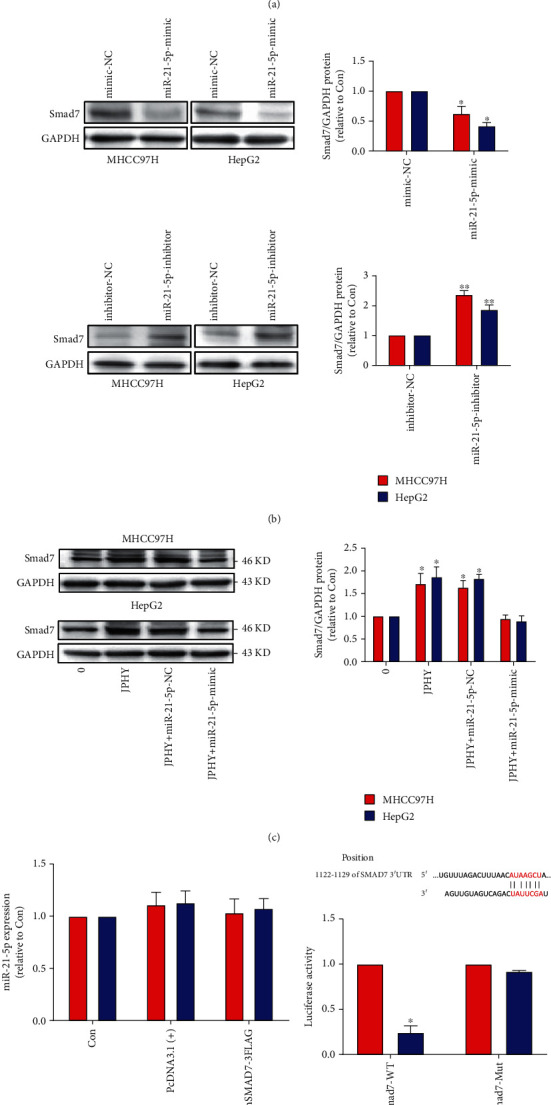
JPHYD inhibited the level of miR-21-5p and increased the Smad7 protein expression in MHCC97H and HepG2 cells. (a) After intervention of JPHYD for 24 hours, we observed the expression of miR-21-5p. (b) After transfection of miR-21-5p mimic and inhibitor into the cells, we observed the expression of Smad7. (c) After transfection of mimic, we added JPHYD to intervene for 24 h and observed the expression of Smad7. (d) After transfection by adding overexpressed Smad7 to the cells, PCR experiment was used to detect the expression of miR-21-5p. (e) The dual luciferase verification report experiment further was verified the relationship between miR-21-5p and Smad7. The asterisk (∗) indicates a significant difference from the control (*P* < 0.05).

The results of qRT-PCR experiments showed that JPHYD significantly reduced the expression of miR-21-5p in MHCC97H and HepG2 cells (Figure 5(a)). In order to further study the relationship between Smad7 and miR-21-5p, we transfected liver cancer cells with miR-21-5p mimics or inhibitors for 48 hours. Then, the western blot analysis was performed. The results showed that the miR-21-5p-inhibitor group significantly upregulated the level of Smad7 protein and the miR-21-5p-mimic group significantly inhibited the level of Smad7 protein (Figure 5(b)). Subsequently, we added JPHYD to the miR-21-5p simulation group and found that JPHYD reversed the decrease of Smad7 protein in the miR-21-5p-mimic group (Figure 5(c)). After transfecting the overexpressed Smad7 plasmid into the cells, PCR experiment showed that the overexpression of Smad7 had no significant effect on miR-21-5p (Figure 5(d)). Subsequently, in order to further determine the relationship between smad7 and miR-21-5p, we conducted a dual luciferase verification experiment. After adding miR-21-5p and wild-type Smad7 reporter vector to the cells, we found that the luciferase activity was significantly inhibited. But after adding miR-21-5p and a reporter vector containing mutant Smad7 to the cells, we found that the luciferase activity was not affected (Figure 5(e)). These findings indicated that miR-21-5p acted as an upstream factor in this process and regulates the expression of Smad7.

### 3.6. JPHYD Inhibited Tumor Growth In Vivo and downregulated the Expression of miR-21-5p, Smad3, N-Cadherin, Vimentin, and Snail and upregulated the Expression of E-Cadherin and Smad7

**Figure 6:**
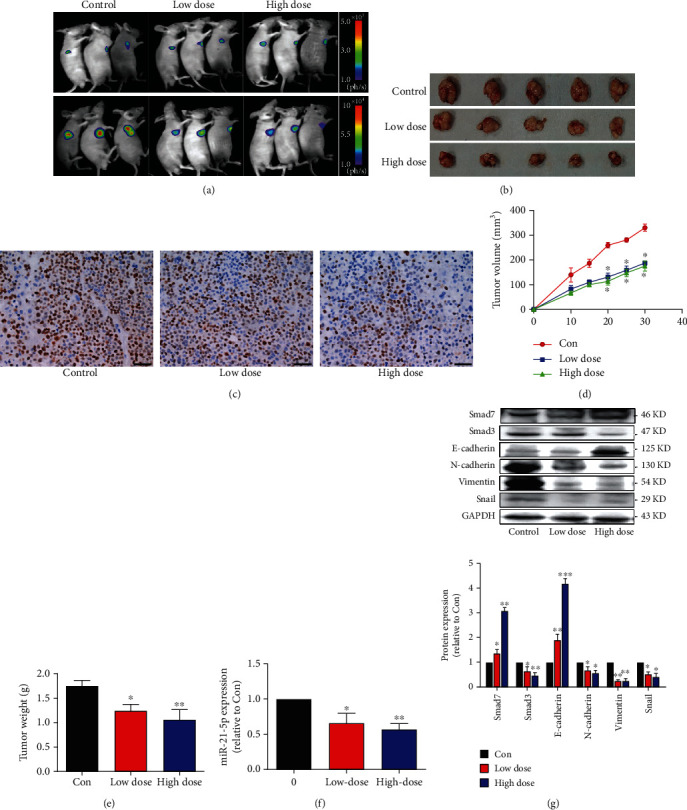
JPHYD inhibited tumor growth and regulated the expression of miR-21-5p, Smad7, Smad3, E-cadherin, N-cadherin, Vimentin, and Snail in vivo. (a) Two different concentrations of JPHYD (low-dose 1 g/mL and high-dose 2 g/mL) were used to treat BALB/c nude mice. By injecting fluorescein substrate into nude mice, the bioluminescence signal was measured on the animal in vivo imaging instrument. (b) Photos of mice and xenografts and (c) anti-ki67 immunostaining in xenograft tumor samples. Scale bar, 50 *μ*m, (d) the size of the tumor, and (e) the weight of the tumor. (f) The expression of miR-21-5p in animal tumor tissues. (g) The expression of related proteins in animal tumor tissues. The asterisk (∗) indicates a significant difference from the control (*P* < 0.05).

We conducted animal experiments to check whether JPHYD inhibits the growth of liver cancer in vivo. We found that JPHYD significantly inhibited tumor growth (Figure 6(a)). The size and volume of tumors in the JPHYD group were smaller than those in the blank group (Figures 6(b), 6(d), and 6(e)). Compared with the blank group, the expression of ki67 decreased in the JPHYD group (Figure 6(c)). In addition, JPHYD significantly inhibited the expression of miR21-5p and the protein levels of Smad3, Vimentin, Snail, and N-cadherin, while E-cadherin and Smad7 proteins increased in tumor tissues treated with JPHYD (Figures 6(f) and 6(g)). These results indicated that both animal and cell experiments showed that JPHYD inhibited EMT in vivo. The results of the in vivo study were the same as those of the in vitro study.

## 4. Discussion

Chinese medicine plays an indispensable role in cancer treatment. Studies have shown that Traditional Chinese Medicine, due to its multifaceted compound composition, can inhibit tumor in a variety of ways, such as promoting tumor cell apoptosis [10] and ferroptosis [11], inhibiting EMT of tumor cells [12], inhibiting tumor angiogenesis [13], and improving immune cell activity [14]. JPHYD is a TCM decoction proposed by our team based on the TCM theory of “ when liver diseases occur, it will spread to the spleen, so the qi of the spleen should be reinforced before it is affected.”

This study provides a new observation on the regulatory mechanism of JPHYD on EMT. Our study found that JPHYD can inhibit the growth and inhibit the invasion and migration of HCC cells. Meanwhile, we found that JPHYD could change the expression of EMT-related proteins (E-cadherin, N-cadherin, Vimentin, and Snail) and Smad-related proteins (Smad7, Smad3, and Zeb1). Subsequently, we found a different expression of miR-21-5p and Smad7 between tumor tissue and nontumor tissue of clinical samples. In addition, we found that JPHY decoction could inhibit the proliferation and migration of HCC cells through miR-21-5p mediating Smad7. Dual luciferase confirms that miR-21-5p is the upstream target gene of Smad7. Moreover, we verified that JPHYD can inhibit the proliferation of HCC cells without obvious systemic toxicity in vivo. Taken together, our results suggest that JPHYD attenuates HCC cell growth, possibly through miR-21-5P mediated EMT and Smad signaling pathways.

The occurrence of distant metastasis of tumors affects the prognosis of cancer patients. Distant metastasis of cancerous cells is a multistep process, including EMT, invading and adhering to local tissues, entering blood vessels, extravasating through blood vessels, and then finally implanting in other tissues or organs. In these processes, EMT is the primary condition for cancerous cell metastasis. E-cad is a key protein for cell adhesion. The lack of E-cad can induce carcinogens activating Wnt, TGF-*β*, HIF-1*α*, and other signaling pathways to promote EMT, which is a reason that EMT affects tumor metastasis [15–18]. In the process of EMT, tumor cells either lack cell junction proteins (such as E-CAD, and ZO1) or increase mesenchymal cell marker proteins (such as N-cad, Vimentin, and Snail) that promote tumor cell metastasis [19]. In recent years, increasing studies have shown that EMT can dynamically change between epithelial phenotype and mesenchymal phenotype and is reversible in promoting tumor cell invasion and metastasis [6, 20, 21]. Liu summarized the correlation between TCM and EMT of tumor and believed that TCM can reverse EMT of tumor [22]. Our study found that JPHYD inhibited tumor proliferation, invasion, and migration through EMT signaling pathway. We also found that JPHYD has a regulatory effect on Smad proteins.

miRNA is a class of noncoding RNAs widely existing in eukaryotes and highly conserved species. Studies have found that miRNAs regulate cell growth, apoptosis, and differentiation by binding to target genes and play important roles in the occurrence and development of tumors. Su et al. [23] proved that miR-21 targeting HBP1 can promote migration, invasion, and EMT of drug-resistant lung adenocarcinoma cells. Besides, miR-21 is highly expressed in liver cancer cells, and inhibiting the expression of miR-21 can inhibit the invasion and metastasis of liver cancer cells [24, 25]. Our study found that the expression of miR-21-5p in tumors is significantly higher than adjacent tissues. Among the Smad proteins regulated by JPHYD, we found that Smad7 is closely associated to miR-21-5p and Smad7 is downregulated in cancer tissues. We verified that miR-21/Smad7 regulates EMT by upregulating the expression of miR-21-5p and downregulating of Smad7 expression in HCC cells. Moreover, we showed that the regulation of miR-21-5p in HCC cells can affect EMT, and JPHYD inhibited EMT by inhibiting miR-21-5p/Smad7 axis in vivo and in vitro. However, due to the lack of further component analysis of JPHYD, further study on the influence of specific components of JPHYD on the regulation of EMT is warranted.

## 5. Conclusion

Our study demonstrated that JPHYD inhibits the proliferation and invasion of hepatocellular carcinoma cells by regulating miR-21-5p/Smad7 and the expression of EMT-related proteins. These results provide a rational of using JPHYD for the treatment of HCC.

## Data Availability

Data available on request can be received from the corresponding author Chong Zhong (email: zhongchong1732@gzucm.edu.cn).
